# Functional validation of *GPIHBP1* and identification of a functional mutation in *GPIHBP1* for milk fat traits in dairy cattle

**DOI:** 10.1038/s41598-017-08668-6

**Published:** 2017-08-17

**Authors:** Jie Yang, Xuan Liu, Dan Wang, Chao Ning, Haifei Wang, Qin Zhang, Li Jiang

**Affiliations:** Key Laboratory of Animal Genetics, Breeding and Reproduction, Ministry of Agriculture & National Engineering Laboratory for Animal Breeding, College of Animal Science and Technology, China Agricultural University, 100193 Beijing, P.R. China

## Abstract

In a previous genome-wide association study (GWAS) on milk production traits in a Chinese Holstein population, we revealed that *GPIHBP1* is a novel promising candidate gene for milk fat content traits. In this study, we performed over-expression and RNAi experiments on *GPIHBP1* in bovine primary mammary epithelial cells. The results showed that the expression of several important milk fat-related genes (*LPL*, *CD36*, *VLDLR*, *ACACA* and *FASN*) increased or decreased when the expression of *GPIHBP1* was up- or down-regulated. To identify the potential functional SNP involved, we explored the genetic variants of *GPIHBP1* and found that a G/A mutation (chr14:2553998) in the promoter region of *GPIHBP1* significantly reduced promoter activity and had an effect on transcription factor binding sites. This finding was consistent with the lower expression of *GPIHBP1* observed in the mammary gland tissue of cows harboring the homozygous AA mutation compared with wild-type homozygous GG or heterozygous AG. Furthermore, association analysis showed that cows with the AA genotype outperformed those with the GG and AG genotypes in terms of the milk fat percentage. Our study demonstrates that *GPIHBP1* could be a strong candidate gene for milk fat content traits and, in particular, the G to A mutation at chr14:2553998 within *GPIHBP1* could be a functional mutation related to its effects.

## Introduction

*GPIHBP1* (glycosylphosphatidylinositol-anchored high-density lipoprotein binding protein 1) is a member of the lymphocyte antigen 6 (Ly6) family^1^. Recent studies showed that *GPIHBP1* plays a critical role in the transportation and localization of lipoprotein lipase (LPL) and serves as a platform for lipolysis in endothelial cells^2–7^. GPIHBP1 also interacts with LPL and represents an important binding site for LPL *in vivo* ^8^. LPL, which is synthesized in the mammary gland and mediates lipolytic processing within the mammary gland, is important for providing lipid nutrients to produce milk fat^9, 10^. Thus, *GPIHBP1* is also involved in the process of milk fat synthesis. The importance of *GPIHBP1* in triglyceride metabolism was demonstrated by Beigneux *et al*.^11^ in mice, who showed that *GPIHBP1*-knockout (*GPIHBP1*−/−) mice displayed severe hypertriglyceridemia, even on a low-fat diet, exhibiting a plasma triglyceride level of 1000–6000 mg/dl at 7–10 weeks of age.

In a previous genome-wide association study (GWAS)^12^ and the subsequent analysis of the novel variants revealed by targeted sequencing of GWAS loci^13^, we found that an SNP in the promoter region of *GPIHBP1* showed a very strong association with the milk fat percentage, with a *P* value of 5.0E-18. We subsequently detected the mRNA expression levels of *GPIHBP1* in eight different tissues of lactating cows. The results revealed that the mRNA expression level of *GPIHBP1* in the mammary gland was much higher than that in the seven other tissues^13^. These results, together with the known biological function of *GPIHBP1* revealed in humans and mice, suggest that *GPIHBP1* is a promising candidate gene for milk fat traits in dairy cattle.

The objective of this study was to validate the effect of *GPIHBP1* on milk fat traits in dairy cattle and to identify the potential functional mutation involved. We investigated the genetic variants of *GPIHBP1* via sequencing, identified four SNPs in the promoter region, and then performed an association analysis of these SNPs and the milk fat percentage in a Chinese Holstein cattle population that was different from the populations used in our previous studies^12, 13^. We subsequently investigated the expressional relationship between *GPIHBP1* and some known milk fat-related genes by over-expressing and down-regulating *GPIHBP1* in bovine primary mammary epithelial cells. To identify the potential functional SNP involved, we tested the effects of the four SNPs on promoter activity via a dual-luciferase reporter system and the effects on transcription factor binding via gel retardation assays. Our results provide valuable information for elucidating the genetic basis of milk fat traits.

## Results

### Exploring genetic variants of *GPIHBP1*

We explored the genetic variants of *GPIHBP1* via sequencing and identified four SNPs (G/A at chr14:2553998, C/A at chr14:2553653, G/A at chr14:2553525, and A/G at chr14:2552574) in the promoter region, one synonymous mutation (C/T at chr14:2550469) in the first exon, one SNP (G/T at chr14:2550326) in the 3′ UTR region, and one SNP (G/C at chr14:2551922) in the third intron. Because knowledge regarding the functions of mutations in introns and synonymous mutations in exons is still limited, and significant SNPs for milk fat traits were found only in the promoter region in our previous work^12, 13^, we focused our attention on the mutations in the promoter region. We genotyped the four SNPs in 158 randomly selected individuals via PCR and performed haplotype analysis using PHASE v2.1.1^14, 15^. The four SNPs were completely linked, and only two haplotypes, GCGA and AAAG, were detected. Among the 158 individuals, 31 were homozygous for the GCGA haplotype, 47 were homozygous for the AAAG haplotype, and 80 were heterozygous.

### Association analysis

An association analysis was performed to test the effects of these SNPs on milk fat and the relationship between their effect and the effect of *DGAT1* K232A. Because these SNPs were completely linked with each other, we analyzed only one of them, chr14:2553998. A total of 6619 cows from a different population than those employed in our previous studies^12, 13^ were used in the analysis. The results showed that when only chr14:2553998 was fit in the model, the *P* value was 1.13E-35. The cows with genotype AA, homozygous for the mutant allele A, exhibited a significantly higher milk fat percentage than cows with genotypes GG and GA (least squares means = 1.24, −10.57 and −4.80, respectively). When fitting only K232A in the model, the *P* value was 8.9E-127, and the least squares means of the three genotypes, AA, GG, and AG, were 17.48, −10.56, and 4.13, respectively. When fitting both SNPs in the model, the *P* values for chr14:2553998 and K232A were 0.0029 and 7.4E-95, respectively. The least squares means for chr14:2553998 were −2.70 (AA), −6.73 (GG), and −4.40 (AG), and those for K232A were 16.75 (AA), −10.36 (GG), and 3.85 (AG). The interaction between the two loci was not significant (*P* > 0.4).

### Haplotype analysis between K232A and chr14:2553998

The haplotype analysis between K232A and chr14:2553998 revealed an linkage disequilibrium (LD) level of *r* ^2^ = 0.137 between these SNPs, and the frequencies of the four haplotypes, G(K232A)-G(2553998), A((K232A)-A(2553998), G(K232A)-A(2553998), and A(K232A)-G(2553998), were 40.6%, 19.7%, 38.2%, and 1.4%, respectively.

### Over-expression of *GPIHBP1* in BMECs

**Figure 1:**
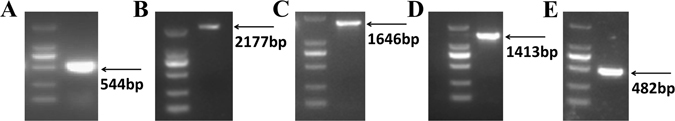
PCR amplification of the open reading frame (ORF) and different promoter segments of *GPIHBP1*. The DNA markers were DL2000:2000bp, 1000 bp, 750 bp, 500 bp, 250 bp, 100 bp. (**A**) Band of the ORF (size = 544 bp). (**B**) Band of the promoter fragment (size = 2177 bp) that contains all four SNPs. (**C**) Band of the promoter fragment (size = 1646bp) that contains the first three SNPs. (**D**) Band of the promoter fragment (size = 1413 bp) that contains the first two SNPs. (**E**) Band of the promoter fragment (size = 482 bp) that contains only the first SNP.

**Figure 2:**
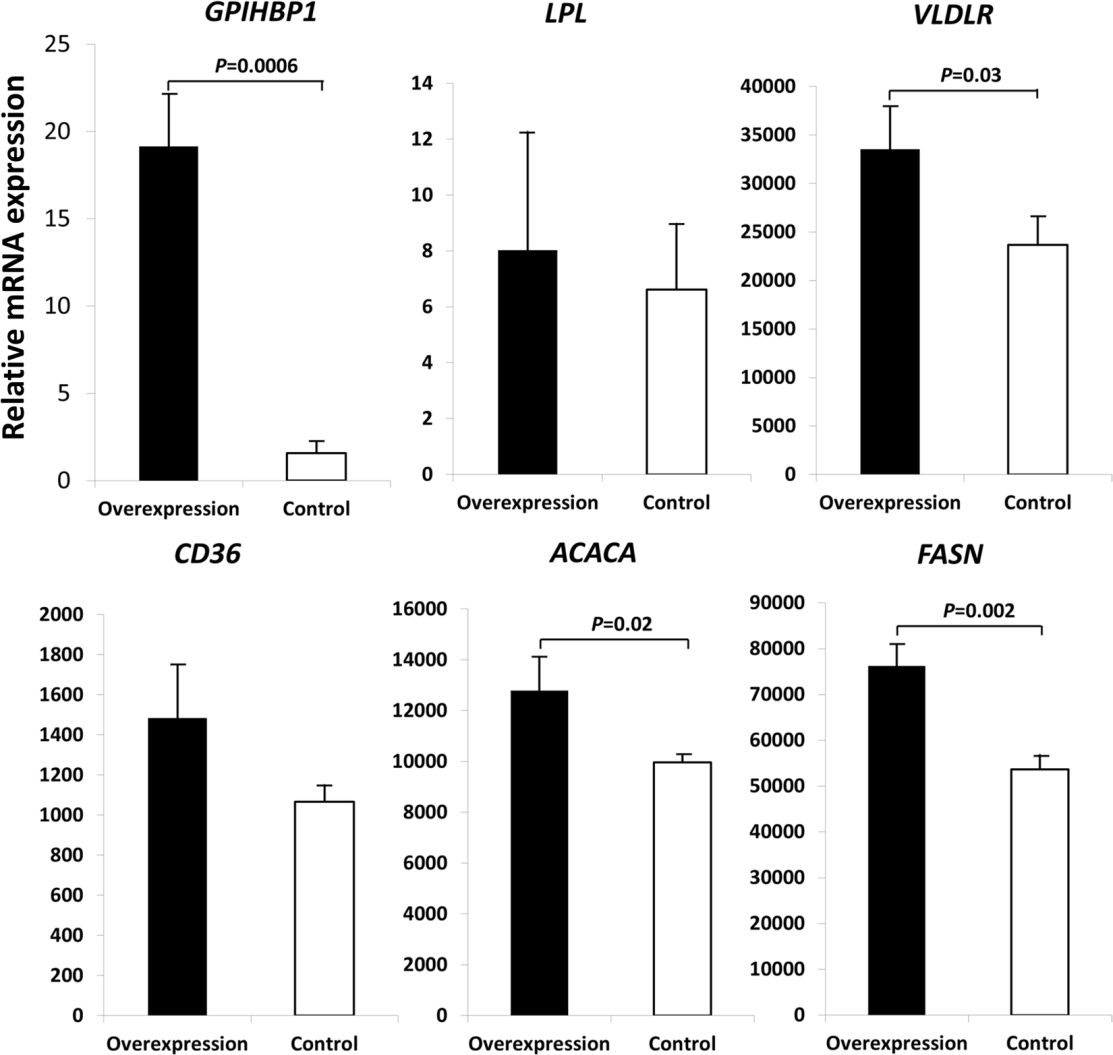
Expression of *GPIHBP1* and five milk fat-related genes in bovine primary mammary epithelial cells that were transfected with the pcDNA3.1(+)-GPIHBP1 eukaryotic expression vector (over-expression) and with an empty vector pcDNA 3.1(+) (control). The vertical axes represent the expression of these genes relative to the expression of the housekeeping gene *GAPDH*.

We constructed a recombinant pcDNA3.1(+)-GPIHBP1 eukaryotic expression vector. The PCR products were loaded into 2% agarose gels, and the band size of the open reading frame (ORF) of *GPIHBP1* was 544 bp (Fig. 1A). After the ORF was inserted into the pcDNA 3.1(+) plasmid, the pcDNA 3.1(+)-GPIHBP1 plasmid sequence was confirmed through DNA sequencing. Then, we transiently transfected the vector into bovine primary mammary epithelial cells (BMECs). After transfection, the mRNA expression level of *GPIHBP1* was analyzed via quantitative RT-PCR. The results showed that the expression of *GPIHBP1* was significantly increased (*P < *0.001) compared with that in control cells (transfected with the empty vector pcDNA 3.1(+)) (Fig. 2). We then detected the mRNA expression levels of five milk fat-related genes (*LPL*, *VLDLR*, *CD36*, *ACACA*, and *FASN*) and found that their mRNA expression levels were all increased (Fig. 2), with the changes in *VLDLR*, *ACACA*, and *FASN* being significant (*P* < 0.05).

### siRNA-mediated silencing of *GPIHBP1* in BMECs

**Figure 3:**
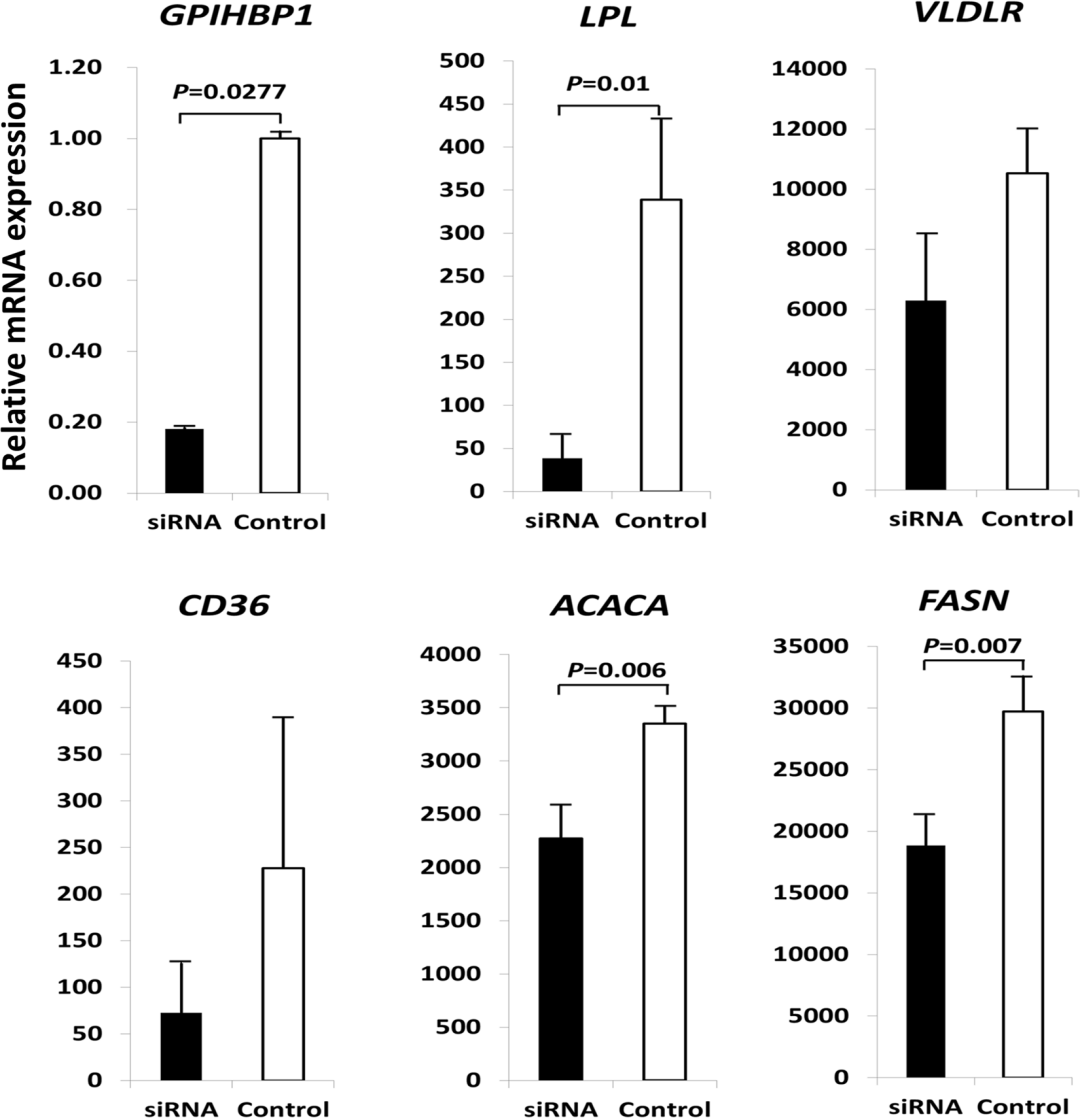
Expression of *GPIHBP1* and five milk fat-related genes in bovine primary mammary epithelial cells that were transfected with Stealth™ RNAi siRNA targeting the bovine *GPIHBP1* gene open reading frame (siRNA) and with the Stealth™ RNAi siRNA Negative Control (control). The vertical axes represent the expression of these genes relative to the expression of the housekeeping gene *GAPDH*.

We transfected BMECs with Stealth™ RNAi siRNA targeting the bovine *GPIHBP1* gene open reading frame. After 48 hours, the *GPIHBP1* mRNA level in BMECs was significantly decreased (*P* < 0.05) compared with that in control cells (transfected with Stealth™ RNAi siRNA Negative Control) (Fig. 3). The mRNA expression levels of the five fat-related genes (*LPL*, *VLDLR*, *CD36*, *ACACA* and *FASN*) were all reduced (Fig. 3), with the changes in *LPL*, *ACACA*, and *FASN* being significant (*P* < 0.05).

### Effects of SNPs on the promoter activity of *GPIHBP1*

**Figure 4:**
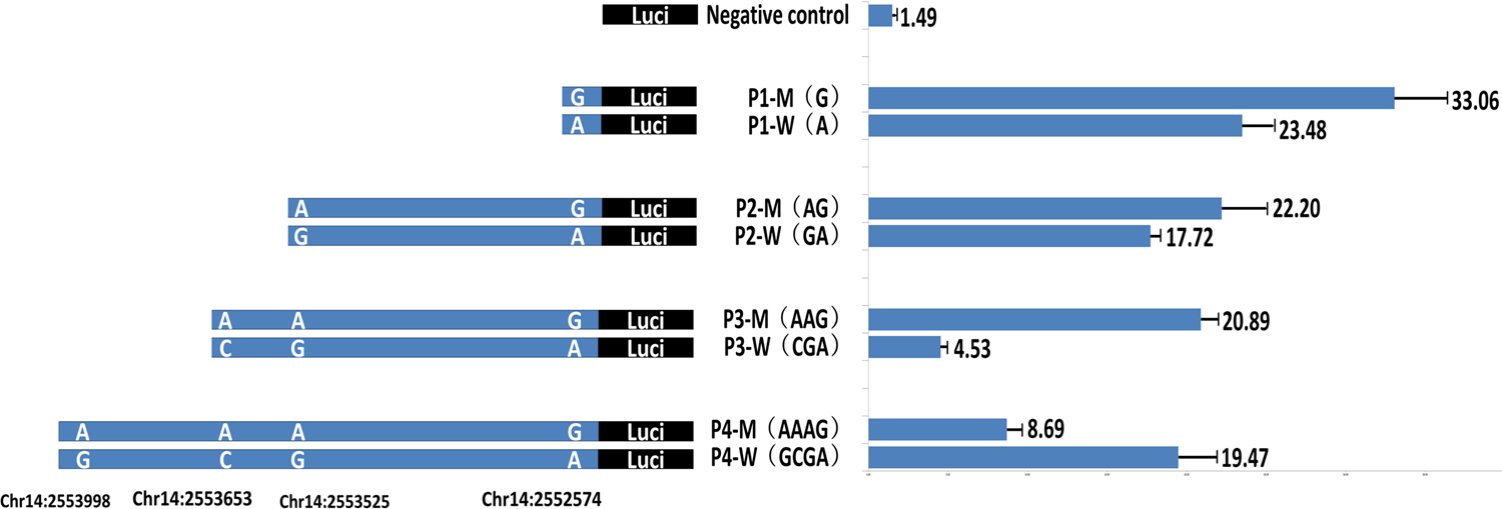
Promoter activity analysis of the bovine *GPIHBP1* gene. There are four completely linked SNPs in the promoter region of *GPIHBP1*. As shown, fragments P1, P2, P3, and P4 contain one, two, three, and four SNPs sites, respectively. We constructed eight types of recombinant promoter vectors with respect to the four fragments and the mutant and wild-type haplotypes. Promoter activities were detected using a dual-luciferase reporter system. The data are expressed as the means and standard errors of three replicates.

To detect the effects of the four SNPs in the promoter region of *GPIHBP1* on promoter activity, we constructed eight recombinant vectors carrying the *GPIHBP1* promoter corresponding to the four SNPs and the wild-type and mutant haplotypes and then measured the promoter activity of different segments using a dual-luciferase reporter system. The band sizes for the *GPIHBP1* promoter fragments corresponding to the four SNPs were 482 bp (P1), 1413 bp (P2), 1646bp (P3), and 2177 bp (P4), respectively (Fig. 1B–E). All the recombinant promoter plasmids exhibited a stronger luciferase response than the negative control (pGL4.14 vector) (Fig. 4), showing the normal promoter activity of the inserted fragments. However, for P1, P2 and P3, the mutant type displayed a stronger luciferase response than the wild type (*P* < 0.01), while for P4, the wild type showed greater luciferase activity than the mutant type (*P* < 0.01). These results suggested that the fourth SNP, G/A (chr14:2553998), might be the functional mutation that significantly reduces the promoter activity of *GPIHBP1*, leading to decreased mRNA expression of *GPIHBP1*.

**Figure 5:**
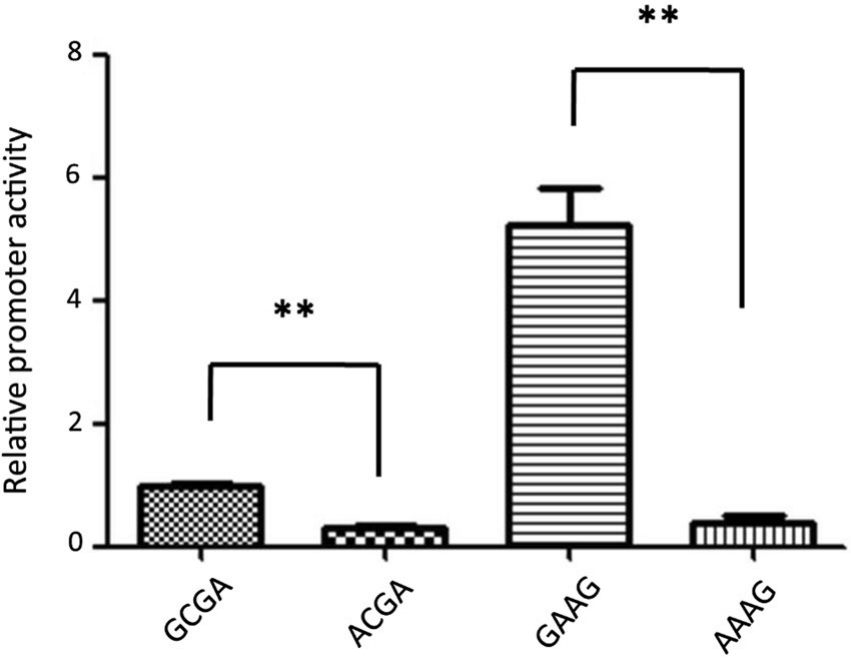
Relative promoter activities of *GPIHBP1* of four different promoter vectors with respect to the four haplotypes (GCGA, ACGA, GAAG, and AAAG). Promoter activities were detected using a dual-luciferase reporter system. The data are expressed as the means and standard errors of three replicates **P < 0.01.

To confirm the causality of the G/A SNP at chr14:2553998 for the decreased mRNA expression of *GPIHBP1*, it is necessary to show its effect on the promoter activity of *GPIHBP1* on the identical haplotypic background for the other three SNPs. Since the four SNPs were completely linked and there were only two haplotypes (GCGA and AAAG) in our experimental population, we reconstituted two haplotypes (ACGA and GAAG) using site directed mutagenesis *in vitro*. Then, we compared the promoter activities of *GPIHBP1* of four different promoter vectors with respect to the four haplotypes (GCGA, ACGA, GAAG, and AAAG). Figure 5 clearly shows that for both haplotypic background AAG and CGA, the mutation G to A at chr14:2553998 significantly reduced (*P* < 0.01) the promoter activity of *GPIHBP1*. These results demonstrated that G to A at chr14:2553998 is the causal mutation leading to the decreased promoter activity of *GPIHBP1*, while the mutations at the other three SNPs increased the activity, which is consistent with the results showing in Fig. 4.

**Figure 6:**
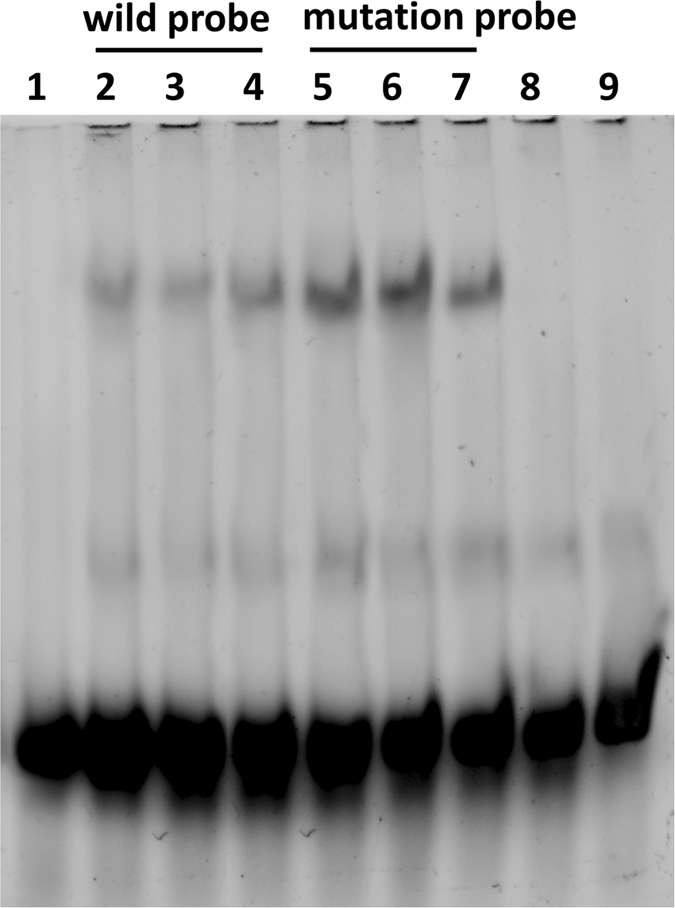
Characterization of the protein binding site for the SNP site, G/A (chr14:2553998), revealed in EMSA experiments. (**A**) Lane 1 = blank; Lanes 2, 3, 4 = nuclear extract + biotin-labeled Probe-wild type; Lanes 5, 6, 7 = nuclear extract + biotin-labeled Probe-mutant type; Lane 8 = nuclear extract + 100 times of cold Probe-wild type; Lane 9 = nuclear extract + 100 times of cold Probe-mutant type. The probes with the mutant allele showed higher nuclear protein affinity than the probe with the wild-type allele.

In addition, we predicted the transcription factor binding sites of the G/A SNP at chr14:2553998 using Genomatix (http://www.genomatix.de/). The results showed that this mutation could influence the binding sites for transcription factors (Table S1). To confirm this prediction, we performed an electrophoretic mobility shift assay. As shown in Fig. 6, without pre-incubation of the nuclear extract with cold probes, protein and labeled probe complexes formed for both the mutant probe and wild-type probe, which retarded the movement of the probe in the gel. However, the nuclear protein showed a higher affinity for the probe with the mutant allele (A) than the probe with the wild-type allele (G), indicating that the mutation had an effect on the binding sites of transcription factors and could indeed regulate the promoter activity of *GPIHBP1*. When the nuclear extracts were incubated in the presence of a 100-fold concentration of the cold probes, protein binding to the labeled probe was abolished, as shown by the lack of visible protein–probe complexes in the gel shift assay.

## Discussion

In a previous study^13^, we revealed a strong association (*P* = 5.0E-18) between the SNP chr14:2553525 (SNP P89 in that paper) in the promoter region of *GPIHBP1* and the milk fat percentage. However, this SNP is only 751 kb away from the K232A mutation in *DGAT1*, which is well known to have major effects on milk fat traits, with a LD level of *r* ^2^ = 0.148. When K232A was fixed in the analysis model, this association became non-significant (*P* = 0.0566), suggesting that this association might be due to LD between chr14:2553525 and K232A. To clarify this issue, we analyzed the association of chr14:2553998 and K232A with the fat percentage in another population with 6619 cows. Because chr14:2553998 is completely linked with chr14:2553525 (as shown in this study), these SNPs should have the same effect on the milk fat percentage. When chr14:2553998 and K232A were separately fitted in the model, both of the SNPs showed a very strong association with the milk fat percentage. When both of them were fit in the model simultaneously, chr14:2553998 still showed a highly significant association, although the *P* value increased markedly, and the interaction between the SNPs was not significant. These results suggest that the SNP chr14:2553998 contributes to the phenotypic variation of the milk fat percentage, although its effect is largely masked by K232A. Haplotype analysis between the two loci revealed that the majority (over 60%) of haplotypes were formed by the favorable (or unfavorable) alleles of the two loci, suggesting that the legitimate effects of *GPIHBP1* and *DGAT1* could be driven by the same haplotype. Moreover, from the point of view of the known biological functions of the two genes, DGAT1 is a key enzyme in triacylglycerol synthesis, catalyzing the final and only committed step in triglyceride synthesis^16^, while GPIHBP1 is a key element in the binding and transport of lipoprotein lipase (LPL)^2–7^, which is essential for providing lipid nutrients to produce milk fat^9, 10^. Thus, both *DGAT1* and *GPIHBP1* play important roles in triglyceride synthesis, either separately or interactively.

To clarify the effect of *GPIHBP1* on milk fat traits, we investigated the effects of over-expressing and silencing *GPIHBP1* on the expression of several milk fat-related genes (*LPL*, *VLDLR*, *CD36*, *ACACA*, and *FASN*). We demonstrated that following the over-expression or silencing of *GPIHBP1*, the mRNA expression of all of these genes was either increased or decreased. The GPIHBP1 protein localizes to the membrane of epithelial cells, where it can bind with LPL and chylomicrons, providing a platform for the lipolysis of triglyceride in chylomicrons by LPL^17–20^. The released non-esterified fatty acids (NEFAs) will be transported into cells by CD36 and VLDLR and used as substrates of ACACA and FASN, both of which are important enzymes in fatty acid synthesis^17, 21–23^. When the expression of *GPIHBP1* is decreased, the binding of LPL with GPIHBP1 is also decreased, such that the process of triglyceride lipolysis in mammary epithelial cells is inhibited, leading to increased accumulation of triglycerides and, thus, a higher milk fat content. Additionally, the reduced release of NEFAs will lead to decreased expression of CD36 and VLDLR. In addition, the expression of ACACA and FASN will also decrease due to the reduction of NEFAs. Our findings following siRNA knockdown *GPIHBP1* as well as over-expression of *GPIHBP1* were fully consistent with these analyses.

**Figure 7:**
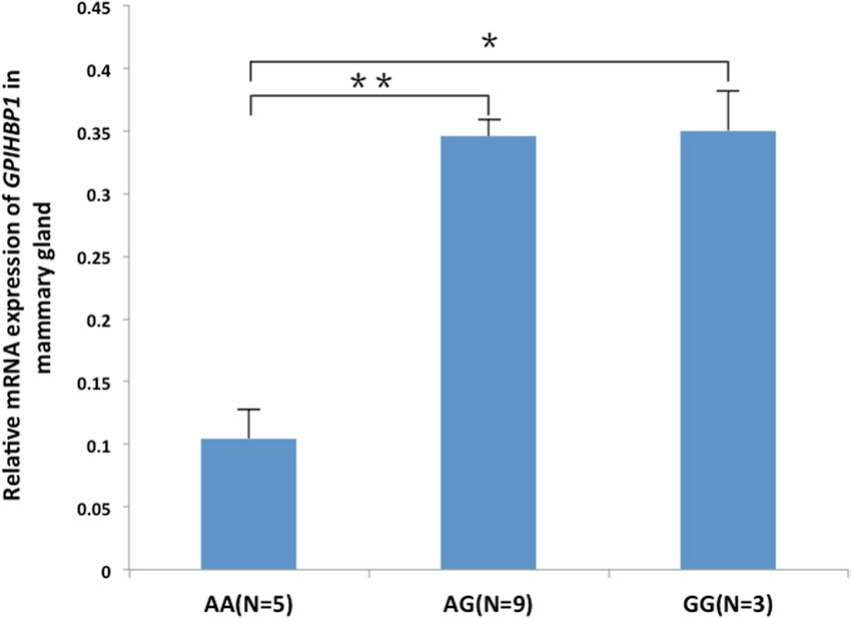
Expression of *GPIHBP1* in the mammary gland of cows with genotypes AA, GG, and GA.

Four SNPs were identified in the promoter region of *GPIHBP1*, which were completely linked with each other. Promoter activity analysis using recombinant promoter vectors revealed that all four SNPs appeared to have effects on promoter activity (Fig. 4). Interestingly, however, the fourth SNP (chr14:2553998), showed an opposite effect compared with the other three SNPs; i.e., the mutant allele of this SNP significantly reduced promoter activity, while the mutant alleles of the other three SNPs increased promoter activity. Through transcription factor binding site prediction and electrophoretic mobility shift assays, we found that the mutation at chr14:2553998 affected binding sites for transcription factors and might regulate the transcript expression level of the *GPIHBP1* gene. To test this possibility, we performed qPCR analysis to detect the expression levels of different genotypes of the SNP chr14:2553998 in the mammary glands of 17 cows. The results showed that the expression of the homozygous mutant (AA) was significantly lower than that of the other two genotypes (Fig. 7), which is consistent with the observation that the mutant allele of this SNP reduced promoter activity. To verify the causality of this mutation for the reduction of the *GPIHBP1* expression and whether its effect is confounded by the genotypes of the other three SNPs, we compared the promoter activities of *GPIHBP1* with varied alleles (G and A) at this SNP on identical haplotypic background (either AAG or CGA) at the other three SNPs. It turned out that with both haplotypic backgrounds the mutant allele A significantly reduced the promoter activity of *GPIHBP1* compared to allele G. Thus, we believe that the chr14:2553998 SNP should be the causal mutation responsible for the reduction of *GPIHBP1* expression. Moreover, the association analysis between the genotypes of this SNP and the milk fat percentage also showed that cows with genotype AA exhibited a higher milk fat percentage than cows with genotypes GG and GA (*P* < 0.001). The effect of this SNP on the milk fat percentage could be caused by the decreased mRNA expression of *GPIHBP1*.

In conclusion, we showed that among the four completely linked SNPs identified in the promoter region of *GPIHBP1*, the G to A mutation at chr14:2553998 could cause the reduction the promoter activity of *GPIHBP1*, leading to decreased expression of *GPIHBP1*. We also showed that the decreased expression of *GPIHBP1* could lead to decreased expression of *LPL*, the gene encoding the LPL enzyme responsible for triglyceride lipolysis. Thus, the higher milk fat percentage associated with the mutant A allele of this SNP could be explained by reduced triglyceride lipolysis and increased accumulation of triglyceride. Therefore, *GPIHBP1* could be a functional gene for milk fat content, and in particular, the G to A mutation at chr14:2553998 in the promoter region of *GPIHBP1* could be a functional mutation for its effects on milk fat content.

## Methods

### Animals

Seventeen Chinese Holstein cows in the same period of lactation were selected from the Beijing Sanyuan Dairy Farm Center. All of the cows were fed under consistent environmental conditions. Mammary gland tissues were collected from each cow within 30 min after slaughter and stored in liquid nitrogen. The entire procedure for the collection of the tissue samples from all animals was performed in strict accordance with the protocol approved by the Animal Welfare Committee of China Agricultural University (Permit number: DK996).

### Culture of BMECs and 293 T cells

BMECs were plated in serum-containing medium DMEM-F12 supplemented with 10% fetal bovine serum (FBS) (Invitrogen, USA), 1% Insulin-Transferrin-Selenium-Sodium Pyruvate (ITS-A) (Invitrogen, USA), 10 ng/ml of epidermal growth factor and 100 units/ml penicillin and streptomycin. The digested cells were cultured at 37 °C in a humidified atmosphere containing 5% CO_2_.

293 T cells were plated in the following serum-containing medium: DMEM supplemented with 5% FBS (Invitrogen, USA) and 100 units/ml penicillin and streptomycin. The digested cells were cultured at 37 °C in a humidified atmosphere containing 5% CO_2_.

### RNA extraction and reverse transcription

Total RNA was extracted from mammary gland tissues or cells using Trizol (Invitrogen, USA), and each sample was reverse transcribed to cDNA in a 40 µL reaction using the Prime Script RT reagent Kit (Takara Biotechnology, Tokyo, Japan).

### Construction of the recombinant pcDNA 3.1(+)-GPIHBP1 eukaryotic expression vector

According to mRNA sequence data for the bovine *GPIHBP1* gene, a pair of specific primers containing Nhe I and EcoR I restriction enzyme cutting sites was designed for amplification of the ORF of the bovine *GPIHBP1* gene using Oligo 6.0 software (forward 5′-CTTTgctagcCCCCGTAGGATGAAGGCA-3′ and reverse 5′-AGGgaattcTCAGAGCCCCATCTCCTG-3′). PCR was performed under the following conditions: after denaturation at 95 °C for 5 min, DNA amplification was performed for 35 cycles at 95 °C for 30 s, 61 °C for 30 s, and 72 °C for 1 min, with a final extension at 72 °C for 7 min. The obtained PCR product was purified using the Omega E.Z.N.A. cycle Pure Kit (Omega, USA).

Plasmid pcDNA 3.1(+) was purchased from Life Technologies (USA) and was transformed into *E.coli* DH5α competent cells (Tiangen Biotech, China) for amplification. Then, the pcDNA3.1(+) vector was isolated from transformants using the AxyPrep Plasmid Miniprep Kit (Axygen, USA). Next, the pcDNA 3.1(+) eukaryotic expression vector and the purified PCR production were digested with the Nhe I and EcoR I restriction enzyme at 37 °C for 4 h. After purification of the digestion products using the Omega E.Z.N.A. Cycle Pure Kit, bovine *GPIHBP1* cDNA was ligated into the pcDNA3.1(+) eukaryotic expression vector using T4 DNA ligase (NEB, USA). Recombinant pcDNA 3.1(+)-GPIHBP1 was amplified in *E. coli* DH5α competent cells (Tiangen Biotech, China) and isolated with the AxyPrep Plasmid Miniprep Kit (Axygen, USA). The correct sequence of the pcDNA3.1(+)-GPIHBP1 plasmid was verified via restriction enzyme mapping and DNA sequencing.

### Transfection of pcDNA 3.1(+)-GPIHBP1 into BMECs

After transformation into *E. coli* DH5α competent cells, pcDNA 3.1(+)-GPIHBP1 and pcDNA 3.1(+) were isolated with the E.Z.N.A. Endo-Free Plasmid Mini Kit I (Omega, USA). The cells were passaged and plated in 6-well plates for 24 hours before transfection at 80% to 90% confluence. The cells were divided into two groups: a transfection reagent+pcDNA3.1(+) (Control) group and a transfection reagent+pcDNA 3.1(+)-GPIHBP1 group (Over expression). The recombinant pcDNA 3.1(+)-GPIHBP1 eukaryotic expression vector or the pcDNA3.1(+) plasmid was transiently transfected into bovine primary mammary epithelial cells according to the instructions for the Roche X-treme GENE HP DNA transfection reagent. Transfection was performed using a 12:1 ratio of the Roche X-treme GENE HP DNA transfection reagent (Roche, USA) (µl) to the vector (µg). At 36 hours after transfection, the cells were collected to detect *GPIHBP1* mRNA expression levels via real-time quantitative PCR.

### Silencing of *GPIHBP1* via RNAi in BMECs

Stealth™ RNAi siRNA (GACGGAUCUCUGACGACCAUAUCCU) targeting the bovine *GPIHBP1* gene open reading frame was designed using BLOCK-iT™ RNAi Designer (Life Technologies, USA) and synthesized by Life Technologies (USA). The Stealth™ RNAi siRNA negative control (Med GC) was purchased from Life Technologies (USA) and used as a control for sequence-independent effects.

One day prior to transfection, cells were seeded without antibiotics and exhibited a density of 80% at the time of transfection. GPIHBP1-siRNA or the Stealth™ RNAi siRNA Negative Control was transfected into bovine primary mammary epithelial cells using the Roche X-treme GENE siRNA Transfection Reagent (Roche, USA) according to the manufacturer’s instructions. Transfection was performed using a 10:1 ratio of the X-treme GENE siRNA Transfection Reagent (µl) to siRNA (µg). Cells were harvested at 48 hours after transfection for mRNA analysis via real-time quantitative PCR.

### Real-time quantitative PCR (qPCR)

The expression levels of *GPIHBP1* and several other milk fat-related genes (*LPL*, *CD36*, *VLDLR*, *ACACA*, and *FASN*) were detected using real-time quantitative PCR. qPCR primers were designed using the Primer 3 web-tool (http://frodo.wi.mit.edu/primer3/) and Oligo 6.0 software and are shown in Table S2. All qPCR procedures were performed using LightCycler 480^®^ SYBR Green I Master on a Roche LightCycler 480^®^ instrument. Real-time PCR amplification was performed in a 96-well plate in a total volume of 20 µL containing the following reagents: 1 µL of cDNA, 1 µL (10pM/µL) of both the forward primer and reverse primer, 10 µL of Blue-SYBR-Green Mix (2×) and water (Roche Applied Science, USA). All qPCRs for each sample were performed in triplicate, and the relative mRNA expression levels were normalized to the housekeeping gene glyceraldehyde phosphate dehydrogenase (*GAPDH*) via the 2^−ΔΔCT^ method^24^.

### SNP detection

Seven pairs of primers (see Supplementary Table S3) were designed to investigate the SNPs of *GPIHBP1*. These primers covered all exons, introns, and 3-kb upstream sequences (as promoter region) of *GPIHBP1*. DNA was extracted from semen samples from 13 bulls. PCR was then performed for each sample, and the obtained PCR products were sequenced using ABI3730XL. All SNPs were detected based on comparison with the reference genome using the DNAMAN software (Lynnon BioSoft, QC, Canada).

### Measurement of *GPIHBP1* promoter activity

#### Amplification of *GPIHBP1* promoter regions

We detected four SNPs in the promoter region of the bovine *GPIHBP1* gene, which were completely linked with only two haplotypes in the test population. Based on this finding, PCR primers (Table S4) containing Kpn I and Bg1 II restriction enzyme cutting sites were designed to amplify four different bovine *GPIHBP1* gene promoter fragments, designated P1 (−509 to −49), P2 (−1440 to −49), P3 (−1673 to −49), and P4 (−2204 to −49), which contained one, two, three, and four SNP sites, respectively. PCR was performed under the following conditions: after denaturation at 95 °C for 5 min, DNA amplification was performed for 38 cycles at 95 °C for 30 s, 70 °C for 1 min, and 72 °C for 2 min, with a final extension at 72 °C for 7 min. The PCR product was purified with the Omega E.Z.N.A. cycle Pure Kit (Omega, USA). Next, the PGL4.14 vector was transformed into *E. coli* DH5α competent cells for amplification and then isolated using an AxyPrep Plasmid Miniprep Kit (Axygen, USA).

#### Construction of recombinant promoter vectors

PGL4.14 and the purified PCR products were digested with the Kpn I and Bg1 II restriction enzyme at 37 °C for 4 h. After purification of the digested products using the Omega E.Z.N.A. Cycle Pure Kit, the *GPIHBP1* promoter fragments were ligated into the PGL4.14 vector using T4 DNA ligase (NEB, USA). The recombinant vectors were amplified in *E. coli* DH5α competent cells and isolated with the AxyPrep Plasmid Miniprep Kit. Finally, restriction enzyme mapping and DNA sequencing were performed to determine the correct sequences of the eight types of recombinant promoters (P1-Wild, P1-Mutation, P2-Wild, P2-Mutation, P3-Wild, P3-Mutation, P4-Wild, and P4-Mutation).

#### Luciferase reporter assay

After transformation into *E. coli* DH5α competent cells, PGL4.14, pRL-TK and the eight types of recombinant promoter vectors were isolated with the E.Z.N.A. Endo-Free Plasmid Mini Kit I (Omega, USA). Then, 293 T cells were cultured in 24-well tissue culture plates overnight, and transfections were performed using Lipofectamine™ 2000 (Life Technologies, USA). The cells were divided into nine groups: PGL4.14 + pRL-TK + Lip2000 (Negative control), P1-Wild + pRL-TK + Lip2000 (P1-W), P1-Mutation + pRL-TK + Lip2000 (P1-M), P2-Wild + pRL-TK + Lip2000 (P2-W), P2-Mutation + pRL-TK + Lip2000 (P2-M), P3-Wild + pRL-TK + Lip2000 (P3-W), P3-Mutation + pRL-TK + Lip2000 (P3-M), P4-Wild + pRL-TK + Lip2000 (P4-W), and P4-Mutation + pRL-TK + Lip2000 (P4-M). According to the manufacturer’s instructions of the Luciferase Reporter Assay system, transfections were performed using a 9:1 ratio of PGL4.14 or the recombinant promoter vector (µg) to the pRL-TK vector (µg) and repeated at least three times for each construct. At 24 hours after transfection, the cells were harvested, and firefly and Renilla luciferase activities were analyzed. To perform normalization for the transfection efficiency, the firefly luciferase value was divided by the Renilla luciferase value for the same sample. All assays were repeated at least three times.

#### *In vitro* experiments

The ACGA and GAAG promoter vectors were generated from P4-W(GCGA) and P4-M(AAAG) by creating one point mutations using QuikChange^®^ Site-Directed Mutagenesis Kit (Stratagene, CA, USA) according to the manufacturer’s instructions. Two pairs of primers were designed for site-directed mutagenesis (see Supplementary Table S5). All changes were confirmed by sequencing. The promoter activities of plasmids with four different haplotypes (GCGA, ACGA, AAAG and GAAG) were measured using a dual-luciferase reporter system and the experimental procedure was the same as described in luciferase reporter assay.

### EMSA (electrophoretic mobility shift assay)

The mammary gland tissues of three cows were used to isolate nuclear protein. Oligonucleotide probes were labeled with biotin at the 5′ends. For competition assays, non-biotin-labeled oligonucleotides were used as cold probes and were added to the binding reaction. The sequences of the double-stranded oligonucleotides used for electrophoretic mobility shift assays (EMSAs) are listed in Table S6. The EMSAs were performed using Viagene’s non-radioactive EMSA kits.

### Association analysis

For the association analysis between the identified *GPIHBP1* SNP or the K232A mutation in *DGAT1* and milk fat percentage, 6619 cows were used. These cows were different from the cows that we previously used for GWAS^12^ and came from 126 sire families. Sequenom MassARRAY iPLEX genotyping technology was employed for the genotyping of both SNPs. The estimated breeding value (EBV) of the cows for the milk fat percentage, which was provided by the Chinese Dairy Association, was then converted to de-regressed proofs (DRP) using the method proposed by Garrick *et al*.^25^. The association analysis was first performed for the two SNPs separately based on the following model: DRP = μ + sire family (random) + genotype (*GPIHBP1* or *DGAT1*) + error. Then, a joint analysis was performed for both SNPs based on the following model: DRP = μ + sire family (random) + genotype (*GPIHBP1*) + genotype (*DGAT1*) + interaction (*GPIHBP1* × *DGAT1*) + error. The SAS procedure GLM was employed for the analysis.

### Haplotype analysis

We genotyped the SNPs detected in *GPIHBP1* in 158 randomly selected individuals via PCR and performed haplotype analysis for these SNPs. In addition, we performed haplotype analysis for the SNP in *GPIHBP1* and the SNP K232A in *DGAT1* in the 6619 cows. The software PHASE v2.1.1^14, 15^ was used for the analysis.

## Electronic supplementary material

Supplementary Tables
