# Supplementary material for: Functional validation of GPIHBP1 and identification of a functional mutation in GPIHBP1 for milk fat traits in dairy cattle
Source: Sci Rep. 2017 Aug 17;7:8546. doi: 10.1038/s41598-017-08668-6 (PMC5561204; doi:10.1038/s41598-017-08668-6)
Supplement: Supplementary file 1 — Supplementary Tables [file 41598_2017_8668_MOESM1_ESM.pdf]

Functional validation of *GPIHBP1* and identification of a functional mutation in *GPIHBP1* for milk fat traits in dairy cattle

Jie Yang, Xuan Liu, Dan Wang, Chao Ning, Haifei Wang, Qin Zhang, Li Jiang*

Table S1 Prediction of transcription factor binding sites for the four SNPs in the promoter region of *GPIHBP1*

SNP	Matrix Family	Detailed Family Information	Matrix	Detailed Matrix Information	Strand	Matrixsim	Sequence*
G	V$TEAF	TEA/ATTS DNA binding domain factors	V$TEAD.01	TEA domain-containing factors, transcriptional enhancer factors 1,3,4,5	(-)	0.951	cggCATTcctctg
G	V$STAF	Selenocysteine tRNA activating factor	V$ZNF76_143.01	ZNF143 is the human ortholog of Xenopus Staf, ZNF76 is a DNA binding protein related to ZNF143 and Staf	(+)	0.917	agaggaatgccgtCCCAgccggccctggggg
G	V$HDBP	Huntington's disease gene regulatory region binding proteins	V$HDBP1_2.01	Huntington's disease gene regulatory region-binding protein 1 and 2 (SLC2A4 regulator and papillomavirus binding factor)	(-)	0.861	ccagggCCGGctgggacgg
G	V$ZICF	Members of ZIC-family, zinc finger protein of the cerebellum	V$ZIC2.02	Zic family member 2 (odd-paired Drosophila homolog) (secondary DNA binding preference)	(+)	0.9	cgtccCAGCcggccc

A	V$TEAF	TEA/ATTS DNA binding domain factors	V$TEAD.01	TEA domain-containing factors, transcriptional enhancer factors 1,3,4,5	(-)	0.953	tggCATTcctctg
A	V$STAF	Selenocysteine tRNA activating factor	V$ZNF76_143.01	ZNF143 is the human ortholog of Xenopus Staf, ZNF76 is a DNA binding protein related to ZNF143 and Staf	(+)	0.91	agaggaatgccatCCCAgccggccctggggg
A	V$HICF	Krueppel-like C2H2 zinc finger factors hypermethylated in cancer	V$HIC1.01	Hypermethylated in cancer 1	(+)	0.961	gaaTGCCatccca
A	V$ZF05	C2H2 zinc finger transcription factors 5	V$ZFP410.01	Zinc finger protein 410, APA-1	(-)	0.922	cggctgGGATggcat
A	V$HDBP	Huntington's disease gene regulatory region binding proteins	V$HDBP1_2.01	Huntington's disease gene regulatory region-binding protein 1 and 2 (SLC2A4 regulator and papillomavirus binding factor)	(-)	0.842	ccagggCCGGctgggatgg
A	V$ZICF	Members of ZIC-family, zinc finger protein of the cerebellum	V$ZIC2.02	Zic family member 2 (odd-paired Drosophila homolog) (secondary DNA binding preference)	(+)	0.9	catccCAGCcggccc

* The SNP sites were indicated by red letter with underline. The core sequence of the transcription factor binding sites are shown in upper case letters.

Table S2 Sequences of forward (F) and reverse (R) primers for real-time quantitative PCR

Gene Names	Sequences	Amplicon size (bp)
GPIHBP1	F：CAAAGCCATCGTCTCCTCC<br>R：TCAGAGATCCGTCCACCGT	110
LPL	F：ACACAGCTGAGGACACTTGCC<br>R：GCCATGGATCACCACAAAGG	101
CD36	F：GTACAGATGCAGCCTCATTTCC<br>R：TGGACCTGCAAATATCAGAGGA	81
VLDLR	F：GCCCAGAACAGTGCCATATGA<br>R：TTTTCACCATCACACCGCC	103
ACACA	F：CATCTTGTCCGAAACGTCGAT<br>R：CCCTTCGAACATACACCTCCA	101
FASN	F：ACCTCGTGAAGGCTGTGACTCA<br>R：TGAGTCGAGGCCAAGGTCTGAA	92
GAPDH	F：GGTGCTGAGTATGTGGTGGA<br>R：GGCATTGCTGACAATCTTGA	180

Table S3 Sequences of forward (F) and reverse (R) primers for SNP detection of *GPIHBP1*

Primer Names	Sequences (5′-3′)	Amplified fragment length(bp)	Position
F1	CCTAACACTGCTGGGAACCT	2155	Chr14:2554368-Chr14:2554349
R1	GGCTCTGAATCTGTGGGAAG		Chr14:2552233-Chr14:2552216
F2	CCCGTAGGATGAAGGCACT	383/352/344	Chr14:2552171-Chr14:2552154
R2	GTCCTGCTGATGGCTTGAC		Chr14:2550578-Chr14:2550560
F3	CATGCAAAGCCATCGTCTC	388	Chr14:2550759-Chr14:2550741
R3	TCATCACAGGTGCTTCCAGT		Chr14:2550288-Chr14:2550269
F4	GGACATGCAAAGCCATCGT	428	Chr14:2550743-Chr14:2550762
R4	GACAGGAGACAGCGGACAGA		Chr14:2550314-Chr14:2550333
F5	TGGAGGCTGACAGTGAGGAA	628	Chr14:2551369-Chr14:2551388
R5	GGAGGAGACGATGGCTTTG		Chr14:2550737-Chr14:2550756
F6	TCAGAGGGACTGGGAAGGG	397	Chr14:2551668-Chr14:2551687
R6	CAGGGTGAGAAATGAGGGTGT		Chr14:2551269-Chr14:2551289
F7	CCGTAGGATGAAGGCACTG	565	Chr14:2552152-Chr14:2552171
R7	GGACACCTGTAAACCTCCACTA		Chr14:2551584-Chr14:2551605

Table S4 Sequences of forward (F) and reverse (R) primers for PCR amplification of different bovine GPIHBP1 promoter region

Names	Sequences (5′-3′)*	Amplified fragment length (bp)	Position
GPIHBP1-P-F1	TTTGGAggtaccAGTGCTGCTGGGAGAGGG	482	-509—-492
GPIHBP1-P-F2	CGGAggtaccCACTGGCAGGAAAAGGCA	1413	-1440—-1423
GPIHBP1-P-F3	CGGAggtaccCAATCTCTGCTCCAAGTGCTC	1646	-1673—-1653
GPIHBP1-P-F4	CGGAggtaccCCTAACACTGCTGGGAACCT	2177	-2204—-2185
GPIHBP1-P-R	TTTTagatctGGCTCTGAATCTGTGGGAAG		-69—-49

* All of the forward primers contain KpnI restriction site. The reverse primer contains Bg1 II restriction site. The enzyme cutting sites are shown in lower case letters. For each fragment, the same reverse primer was used for amplification.

Table S5 Sequences of forward (F) and reverse (R) primers for site-directed mutagenesis

Names	Sequences(5'-3')*	Mutation Site*
GPIHBP1-mut1-F	CAGAGGAATGCCaTCCCAGCCGGCCCTGGGGGCACTCT	GCGA-ACGA
GPIHBP1-mut1-R	TGGGAtGGCATTCCTCTGCAGTCTCAGCCACCAAGCATC	
GPIHBP1-mut2-F	GAGGAATGCCgTCCCAGCCGGCCCTGGGGGCACTCT	AAAG-GAAG
GPIHBP1-mut2-R	TGGGAcGGCATTCCTCTGCAGTCTCAGCCACCAAGCATC	

*: The mutation sites were indicated by underline..

Table S6 Oligonucleotides used in EMSA experiments

Name of probe	Sequences (5'-3')*
Probe-wild	Biotin-5-GCAGAGGAATGCCGTCCCAGCCGGCCCT-3
Probe-mutation	Biotin-5-GCAGAGGAATGCCATCCCAGCCGGCCCT-3

* The SNP sites were indicated by underline.
